# Housing mobility protects against alcohol use for children with socioemotional health vulnerabilities: An experimental design

**DOI:** 10.1111/acer.14911

**Published:** 2022-09-19

**Authors:** Naomi H. Thyden, Nicole M. Schmidt, Spruha Joshi, Huiyun Kim, Toben F. Nelson, Theresa L. Osypuk

**Affiliations:** ^1^ Division of Epidemiology and Community Health, School of Public Health University of Minnesota Minneapolis Minnesota USA; ^2^ Minnesota Population Center University of Minnesota Minneapolis Minnesota USA; ^3^ New York University Grossman School of Medicine New York New York USA

**Keywords:** clinical trials, epidemiology, learning disabilities, neighborhoods, poverty

## Abstract

**Purpose:**

Neighborhood context may influence alcohol use, but effects may be heterogeneous, and prior evidence is threatened by confounding. We leveraged a housing voucher experiment to test whether housing vouchers' effects on alcohol use differed for families of children with and without socioemotional health or socioeconomic vulnerabilities.

**Trial design:**

In the Moving to Opportunity (MTO) study, low‐income families in public housing in five US cities were randomized in 1994 to 1998 to receive one of three treatments: (1) a housing voucher redeemable in a low‐poverty neighborhood plus housing counseling, (2) a housing voucher without locational restriction, or (3) no voucher (control). Alcohol use was assessed 10 to 15 years later (2008 to 2010) in youth ages 13 to 20, *N* = 4600, and their mothers, *N* = 3200.

**Methods:**

Using intention‐to‐treat covariate‐adjusted regression models, we interacted MTO treatment with baseline socioemotional health vulnerabilities, testing modifiers of treatment on alcohol use.

**Results:**

We found treatment effect modification by socioemotional factors. For youth, MTO voucher treatment, compared with controls, reduced the odds of ever drinking alcohol if youth had behavior problems (OR = 0.26, 95% CI [0.09, 0.72]) or problems at school (OR = 0.46, [0.26, 0.82]). MTO low‐poverty treatment (vs. controls) also reduced the number of drinks if their health required special medicine/equipment (OR = 0.50 [0.32, 0.80]). Yet treatment effects were nonsignificant among youth without socioemotional vulnerabilities. Among mothers of children with learning problems, MTO voucher treatment (vs. controls) reduced past‐month drinking (OR = 0.69 [0.47, 0.99]), but was harmful otherwise (OR = 1.22 [0.99, 1.45]).

**Conclusions:**

For low‐income adolescents with special needs/socioemotional problems, housing vouchers protect against alcohol use.

## Abstract 2

In the 1990’s the Moving to Opportunity study randomized low‐income families to receive housing vouchers vs staying in public housing. For low‐income adolescents with special needs/socioemotional problems at baseline, housing vouchers protected against alcohol use 10‐15 years later.

## INTRODUCTION

Alcohol consumption is common among adolescents. Nationally representative studies estimate that approximately two‐thirds of high school students have used alcohol in the past year and more than a third have been drunk (Miech et al., 2020). The negative health consequences of alcohol use, such as traffic deaths and violence, affect the adolescent and those around them. Moreover, alcohol use can inhibit neurocognitive development during this sensitive developmental stage (Crabbe et al., 2011) and may foster adult alcohol disorders (McCutcheon et al., 2013).

Neighborhood context may be an upstream cause of adolescent drinking behaviors, through pathways such as alcohol norms, ambient advertising, or proximity to outlets that sell alcohol. However, evidence is unclear (Jackson et al., 2014), partly because the supporting evidence relies on predominantly cross‐sectional and observational designs (Osypuk, 2013). Experimental designs, while considered the gold standard for causal inference, are relatively rare for testing upstream determinants of alcohol use like neighborhood context. However, the Moving‐to‐Opportunity (MTO) study is one exception; because of its experimental design, it can provide strong evidence for whether changing housing and neighborhood context, via housing vouchers, compared with an in‐place control group who remained in public housing, affects a health behavior such as alcohol use (Ludwig et al., 2008).

Findings from MTO have shown that household heads (mothers) in MTO experienced beneficial effects when randomized to the low‐poverty neighborhood housing voucher treatment group versus the control group, on such outcomes including mental health (psychological distress, major depression), and physical health including reduced obesity, diabetes risk/glycated hemoglobin, in both the short‐ and long‐term evaluation studies (Ludwig et al., 2011; Nguyen et al., 2015; Orr et al., 2003; Osypuk, Schmidt, et al., 2019; Sanbonmatsu et al., 2011). However, there was striking opposite effect modification of the MTO treatment on multiple health and risky behaviors for the MTO adolescents by gender. For example, adolescent girls randomized to receive the housing voucher experienced reduced alcohol use including binge drinking, reduced risky substance use (marijuana, smoking cigarettes, and alcohol use), and improved mental health 4 to 7 years after randomization, compared with controls in public housing. However, these same outcomes (alcohol use, risky substance use, mental health) worsened for male adolescents in the housing voucher treatment group (vs. controls), as did delinquency for male youth in the treatment voucher group who were older at baseline (Kling et al., 2007; Orr et al., 2003; Osypuk, Joshi, et al., 2019; Osypuk, Schmidt, et al., 2012; Osypuk, Tchetgen, et al., 2012; Schmidt & Osypuk, 2017; Schmidt et al., 2018, 2020, 2021).

Findings from MTO and other social experiments suggest that effects may vary not only by gender, but also by other demographic and health‐related vulnerabilities. For example, research from the MTO interim follow‐up (4 to 7 years after randomization) documented that the MTO treatment effects on health, substance use, and behavioral outcomes varied by age, health, developmental problems, crime victimization, and city. Such effect modification by non‐gender variables was not simply detecting the gender effect modification, since some of the patterns showed harmful or null effects for girls, or beneficial effects for boys, depending on the outcomes; moreover, some effect modification involved higher‐order interactions among multiple subgroups, with complicated patterns (Nguyen et al., 2016; Orr et al., 2003; Osypuk, Joshi, et al., 2019; Osypuk, Tchetgen, et al., 2012; Rudolph et al., 2018; Schmidt et al., 2017, 2018).

In addition to opposite treatment effects by adolescent gender described above, evidence from MTO (Nguyen et al., 2016; Osypuk, Schmidt, et al., 2012; Osypuk, Tchetgen, et al., 2012) and other experimental studies (Ertel et al., 2007; McCormick et al., 2006) have documented that some treatments are less beneficial for vulnerable families who have fewer resources and more stressors. Such families typically experience multiple simultaneous disadvantages, e.g., double jeopardy of multiple levels of poverty exposure, and such stressors may embed more deeply to affect health (accumulation of disadvantage; Acevedo‐Garcia et al., 2008; Hertzman & Power, 2003). This frailty hypothesis suggests effects of deep‐rooted stressors may persist for low‐income households and families from racial/ethnic minority households even after relocating. In these analyses, we broadly define a vulnerability as a baseline measure that might capture a challenging circumstance for families, such as not having a car, having a first child before the age of 18, the mother being enrolled as a student, or a child's health problems (See Table 1 for a full list of baseline vulnerability measures.). More vulnerable families may not be able to take full advantage of the resources newly available to them, in line with evidence that more resourced populations receive more benefit from interventions (Osypuk, Schmidt, et al., 2012; Phelan et al., 2010). For example, having a child with health, behavioral, or learning problems might be a vulnerability because the mother spends extra time and/or money on addressing those issues and therefore does not have as many resources to devote to exploring newly available opportunities in health, education, and employment. As a result, families may resort to alcohol use as a coping mechanism. Because housing vouchers remain the largest source of affordable housing assistance for low‐income populations in the United States (e.g., Housing Choice Vouchers, HCV; National Low Income Housing Coalition, 2016), it is important to test whether vouchers work better for some subgroups assigned to the same treatment, so that modifications to this affordable housing policy can be made to ensure all groups can benefit.

**TABLE 1:** Descriptives of baseline covariates and baseline effect modifiers among mothers and youth, by treatment group

	Mothers					Youth				
	Low poverty treatment group (*N* = 1500)	Voucher‐only treatment group (*N* = 700)	Control Group (*N* = 1100)	All Mothers	Test for imbalance *p*‐value	Low poverty treatment group (*N* = 2000)	Voucher‐only treatment group (*N* = 1500)	Control group (*N* = 1600)	All Youth	Test for imbalance *p*‐value
*Mother baseline effect modifiers (vulnerabilities)*										
Aid to Families with Dependent Children (AFDC) or Temporary Aid to Needy Families (TANF)	0.765	0.736	0.765	0.757	0.3675	0.818	0.815	0.816	0.817	0.9841
Household member was victim of violent crime past 6 months	0.429	0.412	0.412	0.419	0.6777	0.426	0.43	0.411	0.422	0.7917
Streets very unsafe at night	0.497	0.521	0.514	0.51	0.5944	0.5	0.541	0.495	0.51	0.2368
Very dissatisfied with neighborhood	0.473	0.473	0.462	0.47	0.8674	0.479	0.507	0.461	0.481	0.2902
Mother enrolled in school	0.155	0.168	0.162	0.161	0.8081	0.164	0.188	0.181	0.176	0.5117
Mother never married	0.632	0.636	0.648	0.638	0.7418	0.661	0.673	0.69	0.674	0.5121
Mother had first child before age 18	0.235	0.265	0.233	0.243	0.3702	0.273	0.303	0.277	0.283	0.4953
Mother employed	0.262	0.262	0.236	0.254	0.3246	0.228	0.2	0.203	0.212	0.3489
*Child baseline effect modifiers (vulnerabilities)*										
Current health problems require medicine or equipment	0.153	0.182	0.16	0.163	0.3121	0.076	0.086	0.076	0.184	0.6432
Behavioral or emotional problems past 2 years	0.101	0.125	0.103	0.108	0.2695	0.019	0.024	0.024	0.104	0.6316
Learning problem past 2 years	0.189	0.245	0.209	0.211	0.0342	0.039	0.057	0.054	0.199	0.0624
Current health problems that limit activity	0.122	0.144	0.124	0.129	0.3974	0.054	0.051	0.051	0.143	0.8881
Suspended or expelled from school past 2 years	0.144	0.15	0.13	0.141	0.5029	0.012	0.017	0.013	0.114	0.4843
School called about behavior in past 2 years	0.287	0.316	0.293	0.297	0.4755	0.074	0.087	0.076	0.273	0.5425
Baseline covariates										
MTO study site										
Baltimore	0.134	0.14	0.135	0.136	0.999	0.119	0.149	0.121	0.128	0.5909
Boston	0.201	0.207	0.205	0.204		0.181	0.179	0.188	0.183	
Chicago	0.205	0.209	0.205	0.206		0.233	0.235	0.226	0.231	
Los Angeles	0.233	0.214	0.226	0.225		0.26	0.216	0.23	0.238	
New York	0.227	0.23	0.229	0.229		0.207	0.221	0.235	0.22	
Parent had a car	0.19	0.19	0.17	0.183	0.4475	0.201	0.193	0.168	0.188	0.2709
Household member had a disability	0.145	0.168	0.148	0.152	0.4458	0.137	0.142	0.15	0.143	0.7714
No teenagers in the home	0.608	0.61	0.646	0.621	0.1669	0.759	0.729	0.768	0.754	0.2571
Household size										
Two	0.223	0.21	0.194	0.21	0.5163	0.097	0.081	0.075	0.086	0.2957
Three	0.302	0.291	0.33	0.308		0.248	0.236	0.257	0.247	
Four	0.233	0.238	0.221	0.231		0.272	0.261	0.242	0.259	
Five or more	0.242	0.261	0.255	0.251		0.383	0.422	0.426	0.408	
Mother wanted to move to escape drugs/gangs	0.786	0.749	0.779	0.773	0.2308	0.771	0.757	0.768	0.766	0.8247
Mother wanted to move for better schools	0.491	0.553	0.481	0.505	0.0192	0.52	0.576	0.494	0.527	0.0166
Mother felt sure they would find an apartment	0.477	0.499	0.456	0.477	0.2757	0.49	0.537	0.477	0.499	0.100
Mother moved more than three times in past 5 years	0.093	0.09	0.108	0.097	0.4103	0.103	0.11	0.123	0.112	0.5339
Mother previously applied for Section 8 voucher	0.4	0.379	0.426	0.402	0.1809	0.36	0.361	0.425	0.382	0.0231
Mother lived in neighbhorhood for 5 years or more	0.599	0.616	0.606	0.606	0.7998	0.536	0.57	0.569	0.557	0.3512
Mother chats with neighbor at least once/week	0.524	0.486	0.549	0.521	0.0669	0.521	0.529	0.532	0.527	0.9072
Mother would talk to neighbor if child got in trouble	0.556	0.521	0.555	0.546	0.3644	0.555	0.535	0.558	0.55	0.7042
Mother had no family in neighborhood	0.64	0.611	0.639	0.631	0.483	0.65	0.609	0.65	0.638	0.2501
Mother had no friends in neighborhood	0.396	0.4	0.409	0.401	0.8442	0.391	0.411	0.408	0.402	0.7125
Baseline age	32.86	33.11	32.84	32.9	0.8161	4.553	4.729	4.706	4.7	0.1572
Mother had GED	0.159	0.183	0.199	0.179	0.049	0.151	0.187	0.219	0.184	0.0431
Mother graduated from highschool	0.381	0.347	0.361	0.365		0.357	0.351	0.34	0.35	
Flag for missing	0.053	0.072	0.074	0.065		0.058	0.059	0.074	0.063	
Race/ethnicity										
Black	0.648	0.629	0.66	0.646	0.6782	0.666	0.66	0.659	0.662	0.7598
Other	0.283	0.283	0.27	0.279		0.279	0.265	0.273	0.273	
Hispanic	0.314	0.34	0.304			0.303	0.304	0.31		
Gender										
Male	0.012	0.022	0.022	0.018	0.1809	0.012	0.025	0.018	0.018	0.2422
Female	0.988	0.978	0.978	0.982	0.8191	0.988	0.975	0.982	0.982	0.7578
*Additional youth‐only covariates*										
Gender										
Male						0.483	0.488	0.513	0.495	0.224
Female						0.517	0.512	0.487	0.505	0.776
Child hospitalized before age 1						0.104	0.107	0.116	0.109	0.6545
Flag for missing						0.032	0.031	0.04	0.034	0.4418
Child weighed less than 6 pounds at birth						0.076	0.086	0.086	0.082	0.5825
Flag for missing						0.048	0.072	0.052	0.056	0.1139
Parent read to child more than once a day						0.147	0.123	0.161	0.145	0.0976
Flag for missing						0.059	0.055	0.05	0.055	0.6571
Gifted student or did advanced coursework						0.047	0.053	0.058	0.052	0.4251
Flag for missing						0.033	0.025	0.034	0.031	0.2994
Flag for Missing: Current health problems that limit activity						0.066	0.076	0.064	0.068	0.606
Flag for Missing: Learning problem past 2 years						0.024	0.016	0.018	0.02	0.2626
Flag for Missing: Suspended or expelled from school past 2 years						0.022	0.022	0.028	0.024	0.6404
Flag for Missing: School called about behavior in past 2 years						0.054	0.067	0.067	0.062	0.2705

*Note*: Means are weighted to account for survey design and family clustering.

Sample sizes are rounded according to FSRDC rounding rules.

All results were approved for release by the U.S. Census Bureau, authorization numbers CBDRB‐FY20‐ERD002‐008 and CBDRB‐FY21‐ERD003‐009.

Additionally, the long‐term follow‐up in this experiment allows testing whether treatment affects the health, including alcohol use, of families over 15 years. Prior work has relied on the 4 to 7 year follow‐up when children were on average age 10 at random assignment; this study leverages the long‐term follow‐up to test whether treatment affects alcohol use among MTO families 10 to 15 years later, when children were much younger (or unborn) at randomization.

Two possible domains of vulnerability that may impact alcohol use include family socioeconomic vulnerability and children's socioemotional health vulnerability. Socioeconomic vulnerability, such as income deprivation, is well documented as affecting parental stress and mental health (Klebanov et al., 1994), parent–child relationships, and adolescent alcohol use (Hardaway & Cornelius, 2014; Leventhal & Brooks‐Gunn, 2000; Swadi, 1999), among other outcomes. However, evidence that children's socioemotional health vulnerability, including children with special needs or special education services, modifies effects of housing on health is sparser.

A small literature has addressed whether special needs children are more or less likely than children in the general population to exhibit adolescent alcohol use, and results are mixed. Some studies find that special education children are less likely to use alcohol compared with the general population, although there is variation across subtype of health disorder (Yu et al., 2008). Others find no association (Cavendish et al., 2012) or find higher risk of substance use among special education children (Kepper et al., 2011).

Aside from the effects on children themselves, parenting a special needs child may be associated with substance use and mental health. Parents of special needs children, particularly of more disabled children, spend more time caregiving and experience increased financial costs for treatment, reductions in employment (Van Dyck et al., 2004), and substantial stress and fatigue (Doig et al., 2009). This often leads to an increase in mental health problems for parents of special needs children, but not necessarily an increase in alcohol use (Cadman et al., 1991). Overall, MTO treatment slightly increased alcohol use among mothers (Sanbonmatsu et al., 2011), which is consistent with the literature that alcohol use increases with higher SES (in this case, where SES is indicated by a housing income supplement or move to a higher SES neighborhood; Grittner et al., 2013).

Leveraging the MTO experimental design, this study tests not only whether the effects of receiving a housing voucher (the treatment) versus remaining in public housing (the control group) affected alcohol use and dependence in youth and mothers 10 to 15 years later, but also whether this voucher treatment effect on alcohol was modified by baseline socioeconomic or socioemotional health vulnerabilities. We hypothesized that the treatment effect of being randomized to a housing voucher (compared with the public housing control group) would be protective against alcohol for families without baseline vulnerabilities and nonsignificant for families with such vulnerabilities.

## METHODS

### Design

The MTO study is a randomized controlled trial of housing vouchers conducted by the Department of Housing and Urban Development in five US cities (Baltimore, Boston, Chicago, Los Angeles, New York; Goering et al., 1999). In 1994 to 1998, over 4600 low‐income families who lived in public housing with children under age 18 volunteered to be randomized to one of three treatment groups. The “low poverty neighborhood” treatment group received housing vouchers to rent a unit in a neighborhood (census tract) where <10% of households were in poverty, plus housing counseling to identify potential rental units. The “Section 8” treatment group was also offered housing voucher, but it was locationally unrestricted. This treatment matches HCV (formerly Section 8) policy (National Low Income Housing Coalition, 2016). The control group received no voucher or intervention but remained eligible for public housing. Randomization ratios varied by each of the five sites and varied over time and were designed to produce sample sizes for primary economic outcomes (Feins & Mcinnis, 2001).

Analyses with MTO has shown that the voucher groups experienced lower exposure to neighborhood poverty over the 15‐year follow‐up period. For example, the control group's average census tract poverty rate over the course of the study (1994 to 2008) was about 40%, while those assigned to Section 8 voucher group lived in census tracts seven percentage points lower for tract % poverty and those assigned to the low‐poverty neighborhood voucher group lived in census tracts nine percentage points lower on % poverty (Sanbonmatsu et al., 2011). Families who adhered to their assigned voucher treatment group experienced even larger neighborhood improvements, with an 11 point (for Section 8) and 18 percentage point (for low‐poverty neighborhood) improvements respectively in tract % poverty compared with the control group, over the course of the study.

At baseline, the household head provided written informed consent, and assent for their children, and completed a survey for themselves and each child in their household (Orr et al., 2003). Because 98% of caregivers in the sample are female, we henceforth refer to the household head as the mother. Follow‐up surveys were collected 4 to 7 years (2001 to 2002) and 10 to 15 years (2008 to 2010) after randomization. Up to three youth per household were sampled. In lieu of following all baseline families, due to financial constraints, only 66% of Section 8 treatment arm families were invited in the final survey sampling frame (Sanbonmatsu et al., 2011), reducing power to detect treatment effect heterogeneity for the Section 8 group. The final samples used for mothers (*N* = 3200) and youth (*N* = 4600) were those who participated in the final surveys, with survey weights used in analyses to account for attrition. Sample sizes were rounded according to requirements for disclosing data from Federal Statistical Research Data Centers (FSRDC).

#### Alcohol outcomes

We analyzed five self‐reported alcohol outcomes among youth ages 13 to 20 at the final survey: (1) ever had an alcoholic beverage, (2) had an alcoholic beverage in the past month, (3) number of days drank alcohol in past month, (4) number of beverages consumed on days they drank, and (5) consumed 5+ alcoholic beverages (binge drinking) on at least one occasion in past month. We also analyzed four self‐reported alcohol outcomes at the final survey for mothers: (1) ever had an alcoholic beverage, (2) had an alcoholic beverage in the past month, (3) number of days drank in past month, and (4) the Severity of Dependence Scale (SDS). SDS is a valid, reliable measure of substance and alcohol dependence (Ferri et al., 2000; Gossop et al., 2002), using a 15‐point scale derived from five items: out of control use, anxiety/worry about missing a drink, worry about use, desire to end use, and difficulty abstaining (Sanbonmatsu et al., 2011). We adopted the cutoff applied by the original MTO investigators, whereby those with scores of 3+ (High SDS) as higher risk of dependence, a cut point consistent with DSM‐IV criteria for substance dependence (Sanbonmatsu et al., 2011).

#### Effect modifiers

Mothers reported baseline child socioemotional health vulnerabilities and family socioeconomic vulnerabilities, which we tested as potential modifiers of the effect of MTO treatment on alcohol outcomes. All effect modifiers were binary variables. Mothers were asked a series of yes/no questions about whether their child had socioemotional health vulnerabilities, including: having a behavioral or emotional problem, learning problem, school called about behavior, and suspension or expulsion from school in the past 2 years; having health problems that limited activity, and health problems that required medicine or special equipment at baseline. In analytic models of youth outcomes, each child‐level effect modifier signifies their own health vulnerability. In analytic models of maternal outcomes, each child‐level effect modifier signifies whether any child in the household at baseline experienced a health vulnerability.

Family socioeconomic and sociodemographic vulnerabilities at baseline included: household head never married, currently receiving welfare, currently unemployed, currently in school, and that a household member was victimized by violent crime (past 6 months). These measures are either known correlates of socioeconomic status (Glymour et al., 2014; e.g., never married) or hypothesized to create an increased cognitive load for the mother (Kawachi, 2014; e.g., in school and household victimization), making them less able to benefit from the voucher‐based relocation. Family socioeconomic/sociodemographic vulnerabilities were reported at the household head level.

These data were analyzed in a FSRDC, which is part of the US Census Bureau, which is an extremely secure environment for accessing and analyzing federal data, including the MTO data. A requirement for disclosing analytic results from a FSRDC is that there are consistent sample sizes across models. We used several strategies to comply with this requirement and produce valid estimates. The original investigators coded baseline variables missing 5% or more as zero and modeled them with a missing indicator, while they imputed those missing less than 5% to the mean based on age, gender, site, and randomization date (Feins and McInnis, 2001). This strategy did not suit our needs analytically because it resulted in binary questions having continuous responses. Therefore, we recoded these continuous mean values to the mode in order to estimate effect modification with binary variables. For example, if the majority of the children in the sample did not need special medicine, the missing children were also recorded as “no,” does not need special medicine. The range of missingness for adult baseline covariates is 0 to 15% and the range of missingness for child baseline covariates is 0 to 16%. There were 10 variables with more than 5% missing and of those, one with more than 10% missing: the sample of youth with behavioral/emotional problems (Sanbonmatsu et al., 2011). In addition, if a child was out of age range for receiving a question, they were coded as “no.” For example, children who were less than 5 years old were not asked about their behavior in school, so for those variables they were retained in the analysis with a “no” response for those questions.

#### Covariates

Because MTO is a randomized trial, measured and unmeasured confounders are anticipated to be balanced across treatment groups, rendering it unnecessary to adjust for baseline covariates to ensure internal validity. However, covariate adjustment helps account for imbalance across treatment groups that may occur by chance and may improve precision if the confounder is associated with the outcome. We used Stata's backward and forward stepwise selection to choose covariates for each outcome (separately) that were associated with the outcome at *p* < 0.2. The variables that were eligible to be included are listed in the “baseline covariates” section in Table 1. We did not get permission from the Census to disclose the full lists of exactly which covariates were included in each model.

### Analysis

We analyzed the main effects of the MTO treatment for each alcohol outcome using intention‐to‐treat (ITT) regression models. ITT analyses retain participants in the group that they were assigned to, whether or not they adhere to the investigator‐assigned exposure. ITT analyses preserve the strength of the randomized exposure in the analysis phase, in lieu of either deleting or reassigning the exposure of observations who did not adhere with the investigator‐assigned treatment, both of which can reintroduce confounding bias that the random assignment is meant to eliminate. ITT is also considered a “pragmatic” approach, which estimates the effect one would expect to observe in the real world when treatments are offered to participants, compared with being conducted in research settings with controlled conditions (Schwartz & Lellouch, 1967). After testing main effects using ITT, we then tested MTO treatment interactions with the proposed baseline socioemotional health and socioeconomic vulnerability modifiers, on alcohol outcomes. We used logistic regression for binary outcomes, and Poisson regression for count outcomes (number of days drank, number of drinks), and exponentiated the effect estimates to compare the voucher treatment versus the public housing control group for each alcohol outcome. For each outcome, we first tested whether the main effect of treatment was homogeneous on alcohol outcomes across the two voucher groups (low poverty, and Section 8), compared with controls. If the homogeneity test was statistically significant (*p* < 0.05), we analyzed the two voucher treatment groups separately, compared with controls, within that outcome model. If the homogeneity test was not statistically significant, then we combined (pooled) the two voucher treatment groups (vs. controls) to increase power. We cautiously interpret estimates with confidence intervals that span 1 when the estimates are part of a larger consistent pattern, and for the Section 8 voucher group, which has a smaller sample size (due to a decision to follow up only 66% of participants at the Final Survey, unlike the other two treatment groups, where 100% of participants were attempted to be followed up) and therefore imprecise estimates.

Next, for every outcome, we tested whether each potential effect modifier generated a significant interaction with MTO treatment. If treatment groups were combined, this was indicated by a significant pooled treatment‐modifier interaction coefficient; if treatment groups were modeled separately, this was indicated by a significant joint test of the low poverty modifier and section 8‐modifier interaction coefficients obtained from Stata postestimation commands. We produced stratum‐specific treatment effects by levels of the effect modifiers using postestimation commands that produce predicted probabilities in Stata 16.1. We present these effects if the interaction tests fell below the threshold *p* < 0.2, as stratum‐specific effects at the *p* < 0.05 level are possible even if the corresponding overall interaction does not meet the *p* < 0.05 threshold. Given considerable gender heterogeneity of treatment effects on health (including alcohol use) at the interim survey (Osypuk, Joshi, et al., 2019), we tested for effect modification of treatment on alcohol use by gender for youth at this final survey, but found no significant gender‐treatment interactions. Each model applied survey weights to account for the design (random assignment ratios), attrition, and within‐family selection of children; additionally, we adjusted standard errors for youth analyses to account for family‐level clustering (Sanbonmatsu et al., 2011). These secondary analyses were conducted under the University of Minnesota's IRB, and all analyses occurred in FSRDCs of the US Census Bureau.

## RESULTS

### Descriptives

Most variables were balanced at baseline across the MTO treatment groups (Table 1), with four exceptions: child had a learning problem (mothers only); mother wanted to move for better schools; maternal education; and mother applied for Section 8 before (youth only).

### Main effects

Table 2 presents the regression results for the main effects of MTO voucher treatment on alcohol use at the final survey, 10 to 15 years after baseline, mothers, and youth. For mothers, the voucher homogeneity test was rejected for lifetime alcohol use, so the two voucher groups were modeled separately there. Opposite our hypotheses, the housing voucher treatment generated harmful effects on alcohol consumption outcomes in main effects models. Compared with public housing controls, mothers in the low‐poverty voucher group exhibited higher (harmful) risk of ever drinking, OR = 1.23 (1.02 to 1.48), while the Section 8 voucher group exhibited effects that were imprecise (possibly from smaller sample size) but seemingly protective (OR = 0.90, 0.72 to 1.13). Mothers in the pooled voucher treatment group exhibited higher risk of alcohol or other drug use problems than controls (RR = 1.44, 95% CI 1.02 to 2.04), with similar adverse but imprecise patterns for past month drinking (OR = 1.07, 95% CI 0.90 to 1.26) and number of days drank in past month (RR = 1.15, 95% CI 0.98 to 1.34).

**TABLE 2:** ITT effects of moving‐to‐opportunity treatment on mother and youth alcohol outcomes at final evaluation by treatment group, compared with control group

	*p*‐value for homogeneity of treatment effect	Low poverty treatment			Voucher‐only treatment			Two treatments combined		
		OR/IRR	LCU	UCI	OR/IRR	LCU	UCI	OR/IRR	LCU	UCI
Mother alcohol use outcomes (*N* = 3200)										
Ever drank alcohol	0.001	1.23	1.02	1.48	0.90	0.72	1.13	–	–	–
Drank in past month	0.17	–	–	–	–	–	–	1.07	0.90	1.26
Alcohol or drug dependent past month	0.22	–	–	–	–	–	–	1.44	1.02	2.04
Number of days drank past month	0.17	–	–	–	–	–	–	1.15	0.98	1.34
Youth alcohol use outcomes (*N* = 4600)										
Ever drank alcohol	0.31	–	–	–	–	–	–	0.89	0.75	1.04
Drank in past month	0.04	0.87	0.72	1.04	1.00	0.83	1.22	–	–	–
Number of days drank past month	0.02	0.93	0.79	1.08	1.05	0.90	1.24	–	–	–
5+ drinks in a day past month	0.02	0.99	0.79	1.24	1.19	0.93	1.52	–	–	–
Number of drinks on days drank	0.21	–	–	–	–	–	–	1.01	0.87	1.17

*Note*: Sample sizes are rounded according to FSRDC rounding rules.

Model results weighted for sampling design and household clustering.

If homogeneity test of two treatment groups *p* < 0.05 then treatment groups analyzed separately, otherwise two treatment groups were combined.

All results were approved for release by the U.S. Census Bureau, authorization number CBDRB‐FY20‐ERD002‐008.

For youth, the voucher homogeneity test was rejected for past month drinking, number of days drank, and binge drinking, so the two voucher groups were modeled separately. Among youth, the voucher treatment main effects were nonsignificant compared with controls, for all alcohol outcomes.

### Effect modification: by children's socioemotional health

**FIGURE 1:**
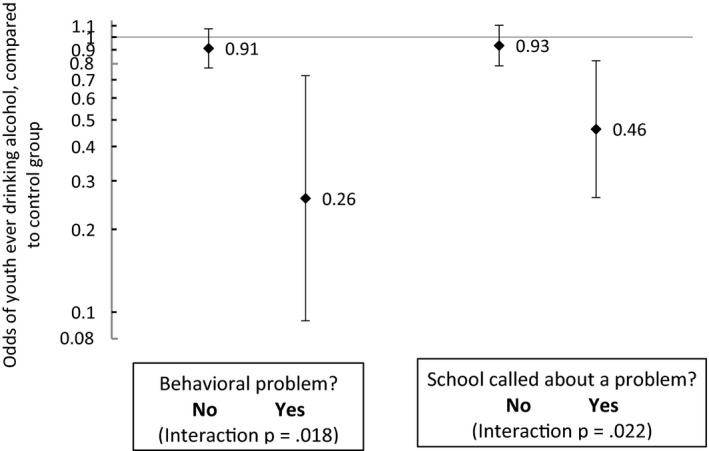
Effect of moving‐to‐opportunity treatment on youth ever drinking alcohol (2008 to 2010), by baseline developmental problem modifiers (1994 to 1998). Model results weighted for sampling design and household clustering. The two voucher treatment groups were combined into a single treatment group, compared to control group. All results were approved for release by the U.S. Census Bureau, authorization number CBDRB‐FY20‐ERD002‐008.

The most consistent significant results across youth and maternal alcohol outcomes emerged for baseline socioemotional health vulnerabilities as effect modifiers of MTO treatment (Table 3). We hypothesized that the MTO voucher treatment would reduce alcohol use among youth without health/developmental (or special needs) problems at baseline. We did detect effect modification; however, it was in the opposite direction as hypothesized; compared with controls, MTO treatment reduced the odds of youth ever drinking alcohol (a protective effect of treatment), only if youth had baseline socioemotional health vulnerability. For example (Figure 1), the MTO Voucher Treatment Odds Ratio for lifetime alcohol use among those with baseline behavior problems was 0.26 (95% CI 0.09, 0.72; treatment–modifier interaction *p* = 0.02); the odds ratio for lifetime alcohol use among children who had baseline problems at school was 0.46 (95% CI 0.26, 0.82; interaction *p* = 0.02). MTO low‐poverty neighborhood treatment (vs. controls) also reduced the number of drinks on days they drank among youth with baseline health problems requiring special medicine/equipment (OR = 0.50, 95% CI 0.32, 0.80; interaction *p* = 0.01; Table S1); treatment was nonsignificant for the Section 8 group (vs. controls). On the other hand, treatment effects were nonsignificant among youth without health/developmental vulnerabilities or special needs. We saw similar, but weaker and less precise, effects for past month drinking and binge drinking. For youth, there were no significant modifiers of MTO treatment on number of days they drank in the past month.

**TABLE 3:** *p*‐values for the interaction between MTO treatment (1994 to 1998) and each baseline effect modifier (vulnerability) for mother and youth alcohol outcomes at the final evaluation (2008 to 2010)

	Mothers (*N* = 3200)				Youth (*N* = 4600)				
	Ever drank alcohol	Drank in past month	Alcohol or drug dependent	Number of days drank past month	Ever drank alcohol	Drank in past month	Number of days drank past month	Number of drinks on days drank	5+ drinks in a day in past month
*Baseline effect modifiers (vulnerabilities)*									
Child socioemotional/developmental									
Behavioral or emotional problems, past 2 years	**0.0599**	0.329	0.151	0.676	**0.018**	0.3213	0.753	0.3397	0.7335
Learning problem, past 2 years	**0.0931**	**0.008**	0.855	0.618	**0.155**	**0.1391**	0.563	**0.1555**	0.3109
School called about behavior, past 2 years	0.6112	**0.183**	0.336	0.813	**0.022**	**0.1591**	0.172	0.9144	0.4337
Current health problems that limit activity	**0.1552**	0.431	0.309	0.154	0.67	**0.1872**	0.713	0.6352	0.9478
Current health problems that require medicine or equipment	0.3147	**0.114**	0.575	0.915	0.221	0.3122	0.63	**0.012**	**0.1809**
Suspended or expelled from school, past 2 years	0.7037	0.59	0.119	0.976	**0.064**	**0.1389**	0.86	0.4182	**0.0662**
Household socioeconomic status									
Mother never married	0.5258	0.487	0.623	0.94	0.977	0.5248	0.808	0.2459	0.1505
Aid to Families with Dependent Children (AFDC) or Temporary Aid to Needy Families (TANF)	0.731	0.753	0.832	0.203	0.834	0.5912	0.642	0.2614	0.7723
Streets very unsafe at night	0.4457	0.903	0.867	0.689	0.677	0.5468	0.342	0.54	0.8633
Very dissatisfied with neighborhood	0.4174	0.351	0.488	0.256	0.883	0.192	0.193	0.3432	0.4601
Mother employed	0.453	0.612	0.488	0.846	0.822	0.429	0.157	0.361	**0.0398**
Mother under 18 years old	0.8661	0.477	0.502	0.77	0.947	0.3335	0.661	0.6037	0.5125
Mother currently in school	**0.043**	0.776	0.646	0.793	0.148	0.5605	0.645	0.6539	0.3352
Household member was a crime victim in past 6 months	0.2543	0.656	0.271	0.173	0.44	0.7436	0.777	0.2189	0.9253

*Note*: Sample sizes are rounded according to FSRDC rounding rules.

Model results weighted for sampling design and household clustering.

If homogeneity test of two treatment groups *p* < 0.05, then treatment groups analyzed separately, otherwise two treatment groups were combined.

Bolded: model included in expanded results in Tables S1 and S2.

All results were approved for release by the U.S. Census Bureau, authorization number CBDRB‐FY20‐ERD002‐008 and CBDRB‐FY21‐ERD003‐009.

**FIGURE 2:**
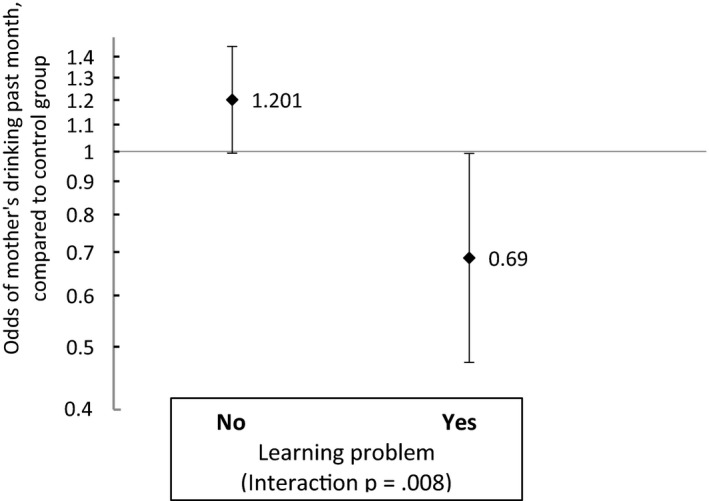
Effect of moving‐to‐opportunity treatment on mother's drinking in the past month at final evaluation (2008 to 2010), by baseline child learning problem modifier (1994 to 1998). Model results weighted for sampling design and household clustering. The two voucher treatment groups were combined into a single treatment group, compared to control group. All results were approved for release by the U.S. Census Bureau, authorization number CBDRB‐FY20‐ERD002‐008.

For mothers' past month alcohol use, one significant modifier emerged (Table 3). For mothers of a child with learning problems, MTO vouchers reduced past month drinking (OR = 0.69, 95% CI 0.47, 0.99), but for mothers without a child with learning problems, MTO vouchers increased past month drinking (OR = 1.22, 95% CI 0.99, 1.45; interaction *p* = 0.01), compared with controls (Figure 2). For lifetime drinking, there were several marginally significant interaction effects (Table 3, 0.05 < = *p* < = 0.20); the patterns on lifetime drinking showed that the low‐poverty neighborhood voucher treatment was harmful in the absence of youth behavioral and learning problems, but Section 8 voucher treatment was beneficial for mothers of youth with these socioemotional vulnerabilities (Table S2). There was no significant effect modification for MTO treatment on substance use problems or past month number of days drank.

### Effect modification: socioeconomic

We found that baseline family socioeconomic vulnerabilities were mostly nonsignificant as potential effect modifiers of mother and youth alcohol outcomes, with the exception of mother's enrollment in school (for mothers' outcomes) and mother's employment (for youths' outcomes; Table 3). Mothers in the low‐poverty neighborhood voucher group who were not enrolled in school at baseline had a higher odds of ever drinking, compared with controls (OR = 1.29, 95% CI 1.05, 1.57; interaction *p* = 0.04), and youth in the low poverty and section 8 voucher groups whose mothers were employed at baseline had a higher odds of binge drinking compared with controls (low poverty OR = 1.66, 95% CI 1.04, 2.65; section 8 OR = 1.53, 95% CI 0.91, 2.57; interaction *p* = 0.04; See Table S3).

## DISCUSSION

Using the MTO study, we probed whether families randomized to receive a housing voucher to subsidize a private market apartment, and move to lower poverty areas, compared with remaining in public housing, experienced different treatment effects on alcohol use outcomes if they did, or did not, have socioemotional health vulnerabilities. Overall, the voucher treatment (compared with controls) had no effect on youth alcohol use 10 to 15 years after randomization, and there were no differences by gender. For mothers, MTO voucher treatment increased alcohol use and drug use problems, compared with the public housing control group. Our analyses did find heterogeneity in the MTO voucher treatment effects on alcohol, by baseline characteristics. Specifically, the effect of MTO treatment on alcohol use a decade later was modified by family socioemotional vulnerabilities, such as a child having developmental problems, health problems, or special needs in school; there was little effect modification by socioeconomic variables. Moreover, the pattern of the treatment effect modification was opposite than hypothesized. We will address these findings in turn.

We did find that family‐level vulnerabilities modified the effect of the MTO voucher experiment on alcohol outcomes 10 to 15 years later, particularly for youth. Children (of both genders) randomized to the MTO voucher group who had socioemotional or special needs at baseline experienced a lower risk of alcohol use—a protective effect—compared with the control group, while children without socioemotional problems at baseline did not experience an effect of MTO voucher treatment on alcohol use. Child‐specific vulnerabilities, including behavioral and emotional problems, learning problems, and the school calling about a child's behavior, mattered the most for modifying voucher treatment on both youth and mother alcohol use. However the pattern of the effect modification was in the opposite direction than we hypothesized, and from that of interim survey results on mental health of older children, such that we thought that families *without* vulnerabilities at baseline would have better outcomes at the final survey, compared with controls (Nguyen et al., 2016; Osypuk, Tchetgen, et al., 2012). Perhaps moving with an MTO voucher was beneficial for vulnerable families because newfound resources benefitted them more. Prior research has shown that families in the MTO treatment groups, especially those who adhered to the treatment and moved, relocated to neighborhoods that were higher quality on a vast array of measured characteristics, including lower poverty, less violent crime, better collective efficacy, more green space (Nguyen et al., 2017; Schmidt et al., 2020, 2021). MTO treatment also shifted the composition of the schools that children attended, toward schools with lower rates of free/reduced lunch and where students were less likely to report feeling put down by teachers than those in the control group (Sanbonmatsu et al., 2011). The services that special needs children receive in their schools, primarily via special education programs, are essential for promoting the learning of children with socioemotional issues, and so it is possible that the change in schools was accompanied an improvement in access to services resulted in reduced alcohol use for the families with socioemotional difficulties.

Socioeconomic vulnerabilities such as being unemployed did not consistently modify the effect of MTO treatment on alcohol at the final evaluation. This aligns with prior evidence from the MTO interim evaluation on adolescent mental health outcomes (Nguyen et al., 2013, 2016). Since all participants in MTO had very low income at baseline, this restricted variability limited power to detect modification by SES in MTO, and thus may explain these nonsignificant findings (Nguyen et al., 2013).

For this sample of youth who were on average 5 years old at baseline, we found that MTO voucher treatment was not associated with alcohol outcomes among youth after 10 to 15 years. There was no effect modification of MTO treatment on alcohol outcomes by gender or by age. This is at odds with the results found for the older cohort of children, who were 10 years old on average at baseline, and 12 to 19 years old when MTO treatment induced beneficial effects on girls' binge drinking, but adverse effects on boys' binge drinking (Osypuk, Joshi, et al., 2019). We believe that the gender effects emerged for the older (earlier) cohort of MTO children due to gender‐specific exposures and adaptations that families make to socialize and protect their children, within the context of high‐poverty neighborhoods. In the case of girls, it seems that relocation to lower‐poverty neighborhoods in the MTO experiment removed the harmful exposures to sexual violence and predation, compared with remaining in high‐poverty neighborhoods, and this may have improved girls' mental health, alcohol use, and a cluster of other risky behavior that adolescent girls may have enacted to cope with such sexual violence (Osypuk, Schmidt, et al., 2012; Popkin et al., 2010). In the case of boys, the socialization that boys receive in order to survive in high‐poverty violent neighborhoods occurs by mid‐childhood and may be difficult to reverse if moving after this point to low‐poverty contexts, where such socialization may be maladaptive and lead to falling to the bottom of the social hierarchy (Anderson, 2000; Caldwell et al., 2010; Osypuk, Tchetgen, et al., 2012). However, for children who moved at considerably younger ages in MTO, they may more easily have reacclimated to a new neighborhood, suggesting a sensitive period in gender‐related child development related to alcohol and other related behaviors (Kuh et al., 2003; Schmidt et al., 2018).

We documented few main effects of MTO treatment, and little effect modification of MTO treatment for the alcohol use and dependence among mothers. There was a harmful effect of MTO treatment on alcohol use among mothers who did not have children with learning problems at baseline, consistent with harmful main effects of MTO treatment on alcohol use among mothers. Alcohol use is higher in higher‐income neighborhoods (Galea et al., 2007), and higher alcohol use is associated with higher socioeconomic status in general (Collins, 2016). It is therefore possible that mothers who moved to lower‐poverty neighborhoods could have been influenced to use alcohol more. It would be helpful for future research to look into differential effects of MTO treatment on other outcomes such as mental health by whether families had children with socioemotional health vulnerabilities. Overall, MTO treatment improved psychological distress but did not affect serious mental illness (Sanbonmatsu et al., 2011).

Our original hypotheses did not anticipate that mothers would exhibit different patterns from their children. However, the sensitive period model of life course theory is consistent with finding stronger effects among adolescents if neighborhood (as an upstream social determinant of health) is more influential for affecting alcohol during childhood, since alcohol use may be more modifiable among youth compared with adults (Hertzman & Power, 2003; Kuh et al., 2003). Alternately, it may be that the child's own socioemotional health might be what's important for patterning their own alcohol use, rather than the alcohol use of their mothers, which is one step removed.

### Limitations

Because the MTO study was originally designed to measure economic outcomes, the original investigators did not include health measures such as alcohol use at baseline. This means we did not have some baseline measures that may be relevant for testing effect modification of treatment on alcohol use for adults such as baseline drinking behavior. The randomized design and our ITT analysis minimizes risk of bias, since even if alcohol use was measured at baseline, it would be expected to have been evenly balanced across treatment groups. However, there was a statistically significant difference between treatment groups in a few covariates at baseline including the mother's education level, which is a known correlate with alcohol use. We adjusted for baseline covariates to lessen the effects of unmeasured variables that were imbalanced across treatment groups. In addition, because we wanted to preserve the analytic benefits of randomization, we did not include postrandomization neighborhood‐level or individual‐level covariates in our analyses that are associated with alcohol use. Our analyses estimated the total effects of the MTO housing intervention, but do not identify pathways or mediators to alcohol use, for example, if the MTO treatment affects the child's use via the mother's use. This is an important direction for future research.

The way we accounted for missingness in baseline vulnerabilities has the potential to create bias in the results. We coded missing values to the mode, which may misclassify some individuals—most likely as false zeroes—and potentially dilute true associations and risk type 2 error. We cannot predict the way missingness in effect modifiers might affect the stratified treatment effect results.

The MTO sample comprises low‐income families living in urban areas and is predominantly Black and Hispanic. These findings may not generalize beyond this population, but are still important for housing policy. Although the MTO treatment was designed to measure the causal effect of neighborhood characteristics through moving to a different neighborhood, the more complicated reality is that for families who moved, the change in neighborhood was not the only change they experienced. For example, moving itself is a stressor for children (Jelleyman & Spencer, 2008). In addition, moving might disrupt established social networks (Waterston et al., 2004) that were beneficial to the family.

The available variables that operationalized child socioemotional vulnerabilities at baseline were asked of mothers in binary questions and were not designed to capture other dimensions of special needs children's health, or special education services; severity of socioemotional disorders is also unknown. Within the category of whether a child required special medicine, there may be several conditions that affect outcomes in different ways. Moving forward, having multidisciplinary teams, including those that advise on the best health measures, may improve the ability of trials manipulating social exposures to inform social epidemiology (Glass et al., 2014).

## CONCLUSIONS

MTO, the Housing Choice Voucher program, and other housing mobility policies expand neighborhood choice for low‐income families by providing subsidies for renting apartments in the private market. Although the primary goal of HCV focuses on income support, the locational aspects of the program can be enhanced to support all families to equally benefit from moving to lower‐poverty and higher‐opportunity neighborhoods. However, HCV and housing mobility policies have substantially higher demand that vastly outstrips supply in the United States (Ellen, 2020). Although housing mobility programs, as compared with simple voucher programs, are generally more comprehensive in addressing multiple barriers and offering a range of resources, they remain very small scale and define housing need narrowly as very low household income. As scholars and practitioners have noted, it is necessary to address structural conditions in the neighborhoods and schools of low‐income families and to assess whether such investments can replicate some of the positive effects MTO had on families and children, and their neighborhoods, by virtue of moving/relocation.

This work exploring the junction between many sectors and fields, such as child development, housing policy, and alcohol outcomes, is consistent with a health in all policies approach (Collins & Koplan, 2009). Because housing vouchers continue to be the main affordable housing policy available to low‐income families (National Low Income Housing Coalition, 2016), it is important to understand the nuances of the MTO study to inform how future housing policies can help all families benefit from expanded housing choice.

## FUNDING INFORMATION

This research was supported by National Institutes of Health grants R21 AA024530 and R01 HD090014, PI Theresa Osypuk, and P2 CHD041023, PI John Robert Warren/Theresa Osypuk. S. Joshi was supported by the NIH/NIDA funded T32 training grant 5T32 DA007233‐36. N. Thyden was supported by the NIH/NICHD‐funded T32 training grant in Population Health T32 HD095134 and R01 HD090014.

## CONFLICT OF INTEREST

None.

## Supporting information


Table S1

Table S2

Table S3
